# Counting the cost of child mortality in the World Health Organization African region

**DOI:** 10.1186/s12889-015-2465-z

**Published:** 2015-11-06

**Authors:** Joses M. Kirigia, Rosenabi Deborah Karimi Muthuri, Juliet Nabyonga-Orem, Doris Gatwiri Kirigia

**Affiliations:** Research, Publications and Library Services Programme, Health Systems and Services Cluster, World Health Organization, Regional Office for Africa, Brazzaville, Congo; Department of Psychology, United States International University (USIU), Nairobi, Kenya; Health Systems and Services Cluster, World Health Organization, Regional Office for Africa, Brazzaville, Congo; KEMRI-Wellcome Trust Research Programme, Nairobi, Kenya

## Abstract

**Background:**

Worldwide, a total of 6.282 million deaths occurred among children aged less than 5 years in 2013. About 47.4 % of those were borne by the 47 Member States of the World Health Organization (WHO) African Region. Sadly, even as we approach the end date for the 2015 Millennium Development Goals (MDGs), only eight African countries are on track to achieve the MDG 4 target 4A of reducing under-five mortality by two thirds between 1990 and 2015. The post-2015 Sustainable Development Goal (SDG) 3 target is “by 2030, end preventable deaths of new-borns and children under 5 years of age”. There is urgent need for increased advocacy among governments, the private sector and development partners to provide the resources needed to build resilient national health systems to deliver an integrated package of people-centred interventions to end preventable child morbidity and mortality and other structures to address all the basic needs for a healthy population. The specific objective of this study was to estimate expected/future productivity losses from child deaths in the WHO African Region in 2013 for use in advocacy for increased investments in child health services and other basic services that address children’s welfare.

**Methods:**

A cost-of-illness method was used to estimate future non-health GDP losses related to child deaths. Future non-health GDP losses were discounted at 3 %. The analysis was undertaken with the countries categorized under three income groups: Group 1 consisted of nine high and upper middle income countries, Group 2 of 13 lower middle income countries, and Group 3 of 25 low income countries. One-way sensitivity analysis at 5 % and 10 % discount rates assessed the impact of the expected non-health GDP loss.

**Results:**

The discounted value of future non-health GDP loss due to the deaths of children under 5 years old in 2013 will be in the order of Int$ 150.3 billion. Approximately 27.3 % of the loss will be borne by Group 1 countries, 47.1 % by Group 2 and 25.7 % by Group 3. The average non-health GDP lost per child death will be Int$ 174 310 for Group 1, Int$ 57 584 for Group 2 and Int$ 25 508 for Group 3.

**Conclusions:**

It is estimated that the African Region will incur a loss of approximately 6 % of its non-health GDP from the future years of life lost among the 2 976 000 child deaths that occurred in 2013. Therefore, countries and development partners should in solidarity sustainably provide the resources essential to build resilient national health systems and systems to address the determinants of health and meet the other basic needs such as for clothing, education, food, shelter, sanitation and clean water to end preventable child morbidity and mortality.

## Background

**Fig. 1:**
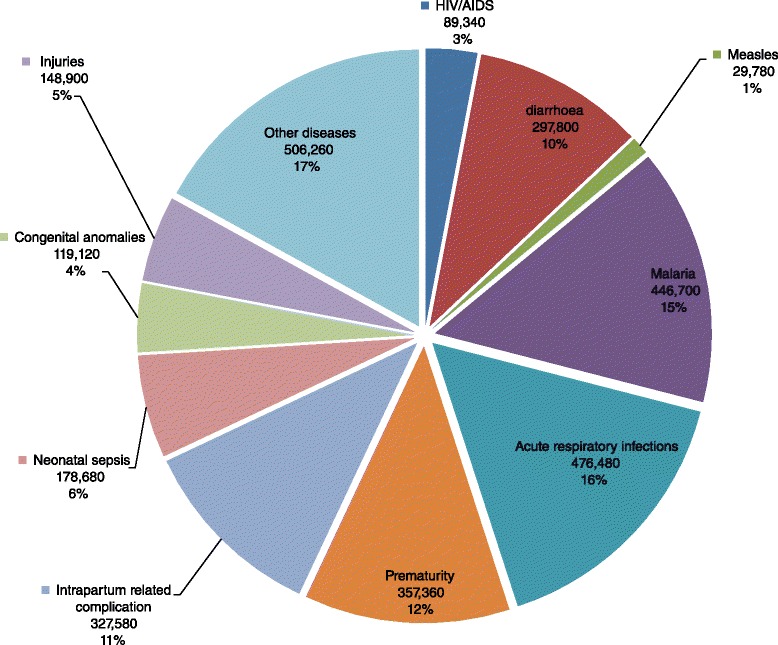
African Region distribution of deaths by cause among children aged less than 5 years (%)

A total of 6.282 million deaths occurred worldwide among children aged less than 5 years in 2013 [1]. About 2.976 million (47.4 %) of these were in the World Health Organization (WHO) African Region (see Fig. 1 for the distribution of the deaths by cause [1]). The top five causes of death, which accounted for 64 % of child mortality included acute respiratory infections (16 %), malaria (15 %), prematurity at birth (12 %), intrapartum related complications (11 %) and diarrhoea (10 %). Basically, child mortality in the Region is mostly due to a few largely preventable causes.

**Fig. 2:**
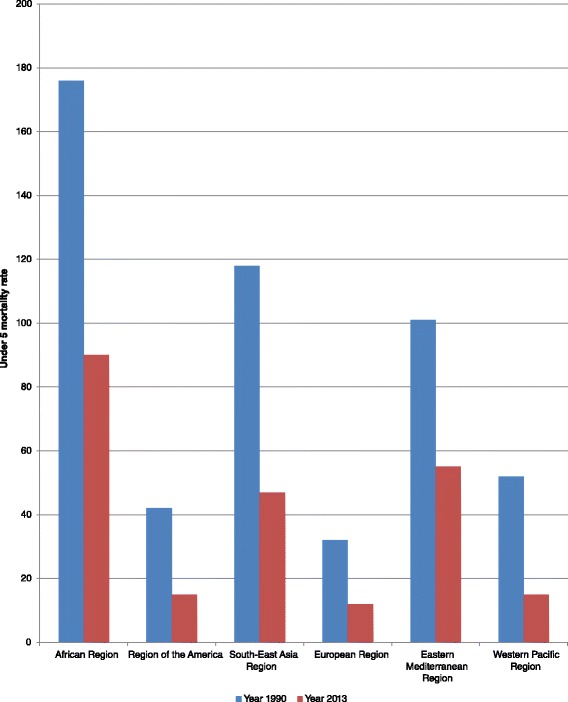
Inter-regional comparison of under-five mortality rate (probability of dying by age 5 per 1000 live births), for 1990 and 2013

The WHO African Region has made some progress in improving child health. For instance, the neonatal mortality rate dropped from 45 per 1000 live births in 1990 to 31 in 2013; the infant mortality rate, which is the probability of dying between birth and 1 year of age per 1000 live births, declined from 106 in 1990 to 60 in 2013; and the under-five mortality rate declined from 176 per 1000 live births in 1990 to 90 in 2013. But under-five mortality in the Region is still far much higher than for all other WHO regions (see Fig. 2) [1].

In spite of the improvement in child health, only eight of the 47 African countries in the WHO Region are on track to achieve MDG4 target 4A on reducing under-five mortality by two thirds between 1990 and 2015. These are Eritrea, Ethiopia, Liberia, Madagascar, Malawi, Niger, Rwanda and Tanzania. Any child death signifies a country’s inability to assure every child of the fundamental human right to the highest attainable standard of health and to life.

The human rights stipulated in Articles 3 and 25 of the 1948 United Nations General Assembly Universal Declaration of Human Rights continue to be violated for the under-fives in African countries and by their development partners. Article 3 indicates that every child has the right to life, liberty and security of person, and Article 25 that every child has the right to a standard of living adequate for health and well-being, including food, clothing, housing, medical care, necessary social services, security and social protection [2]. These rights have been expounded in the Declaration of the Rights of the Child [3], the Convention on the Rights of the Child [4], the African Charter on the Rights and Welfare of the Child [5], and the African Charter on Human and Peoples Rights [6].

Even as children continue to die, cost-effective interventions that could prevent majority of the deaths are available but not accessible to most of those who need them. For instance, 49 % of births are not attended by skilled health personnel, 25 % of neonates are not protected at birth against neonatal tetanus, and among 1-year-olds, 26 %, 25 %, 24 % and 28 % are not immunized with the measles vaccine, DPT3, HepB3 or Hib3, respectively [1]. For under-fives, 41 % of those who need vitamin A supplementation do not receive it, about 51 % of those with acute respiratory infection symptoms are not taken to a health facility, 64 % of those with suspected pneumonia do not receive antibiotics, and 51 % of those with diarrhoea do not receive oral rehydration salts or the recommended home fluids. Thirty-one percent of pregnant women with HIV did not receive antiretrovirals to prevent mother to child transmission of the disease.

In 59 % of African countries fewer than 41 % of under-fives are sleeping under insecticide treated nets (ITNs). Indeed, out of 39 countries with data, ITN coverage was less than 21 % in 6 countries, 21–40 % in 17 countries, 41–60 % in 12 countries, and 61–80 % in 4 countries. Fewer than 41 % of under-fives with a fever received treatment with any antimalarial in 79 % of the countries. Of the 39 countries with data, the treatment coverage was less than 21 % in 10 countries, 21–40 % in 21 countries, 41–60 % in 7 countries, and over 60 % in 1 country [1].

In spite of the numerous child health-related resolutions adopted by the countries and decisions made at various sessions of the United Nations General Assembly [3, 4], the World Health Assembly [7–10], the African Union [11–16] and the WHO Regional Committee for Africa [17–29], significant numbers of children do not have access to health services or services that meet their basic needs such as shelter, education, water, sanitation and security. This is largely because the national health systems and structures for providing these services are weak from gross underinvestment [30, 31]. Cost-of-illness information is needed by the ministry of health for use in advocacy with the ministry of finance for increased fiscal space for health, and with the ministries in charge of other basic services, the private sector and development partners to invest more resources in child health and welfare services to end preventable child morbidity and mortality.

This paper attempts to answer the question: What is the impact of child deaths on expected/future non-health gross domestic product (GDP) in the WHO African Region? The specific objective was to provide an estimate of the expected/future productivity losses from child deaths in the Region in 2013 for use in advocacy for increased investments in services for child health and other basic services that address children’s welfare.

## Methods

### Conceptual framework

Child deaths have a negative impact on future macroeconomic output. They increase health expenditure, cause attrition of future labour and productivity, and erode investments in human and physical capital formation [32]. This study uses the cost-of-illness model to estimate the impact of child deaths on non-health components of future GDP) [33]. GDP is the sum of personal consumption expenditures, gross private investment, government consumption spending and net exports (exports minus imports) [34]. Child deaths reduce future spending on goods and services; future labour force; future household savings, and hence investments; the number of future tax payers, and hence future tax revenues; and the number of future exports producers, bleeding future exports earnings. Since children are not part of the current labour force, their deaths affect future not present flows of GDP.

The non-health GDP loss due to deaths of under-fives in i^th^ country (NHGDPLoss) is the product of the total number of discounted life years above the minimum employment age lost, per capita non-health GDP in purchasing power parity (PPP) and total child deaths. Each country’s discounted total non-health GDP loss due to child deaths was calculated using Eq. 1.

1 $$ \begin{array}{l} NHGDPLos{s}_i={\displaystyle \sum_{t=1}^n\left\{\left[1/{\left(1+r\right)}^t\right]\right.}\times \left[ NHGDPP{C}_{Int\$}\right]\times \left.\left[TCD\right]\right\}=\\ {}\kern2.16em \left\{\left[1/{\left(1+r\right)}^1\right]\right.\times \left[ NHGDPP{C}_{Int\$}\right]\times \left.\left[TCD\right]\right\}+\\ {}\kern2.16em \left\{\left[1/{\left(1+r\right)}^2\right]\right.\times \left[ NHGDPP{C}_{Int\$}\right]\times \left.\left[TCD\right]\right\}+\dots +\\ {}\kern2.16em \left\{\left[1/{\left(1+r\right)}^n\right]\right.\times \left[ NHGDPP{C}_{Int\$}\right]\times \left.\left[TCD\right]\right\}\kern0.24em ......................\end{array} $$

Where: 1/(1 + *r*)^*t*^ is the discount factor; *r* is the rate of discount of future losses; *t* is the first year of life lost, and *n* is the final year of the total number of years of life lost per child death, which is obtained by subtracting the average age at death (AAD) from each country’s average life expectancy at birth; *NHGDPPC*_*Int$*_ is the per capita non-health GDP in purchasing power parity (PPP), which is obtained by subtracting per capita total health expenditure (PCTHE) from per capita GDP (Int$ GDPPC); *TCD* is the total number of child deaths that occurred among the under-fives in country *i* in 2013. The year 2013 was used as the base year to which losses occurring in future years were discounted. The value of the discount factor decreases as one move from the base year into the future years, so losses in successive years have a lower value than similar losses in the first year. The weight (discount factor) applied to the GDP losses of different years then depends not just upon the discount rate, r, but also on the number of years, t, over which the discounting is conducted. When discounting is applied so that all GDP losses are revalued relative to year 0 (i.e., 2013 in this study), the revalued resources are referred to as present values [35].

The average age at death was 2.5 years, i.e. 0 plus 5 years divided by 2. Since according to Article 2 of the International Labour Organization (ILO) convention, the legal minimum age for employment is 15 years [36], the future productive years of life lost equal each country’s life expectancy at birth minus 14 years.

The per capita non-health GDP in purchasing power parity for each of the 47 countries in the WHO African Region was obtained by subtracting per capita total health expenditure from per capita GDP.

#### Illustration of calculation of loss in total non-health GDP

The example below on calculation of child death-related loss in non-health GDP uses actual information on Nigeria:

- Total number of child deaths in Nigeria in 2013 (TCD) = 804000
- Average age at death among under 5 year old children (AAD ), i.e. (0 + 5)/2 = 2.5 years
- Nigeria’s life expectancy at birth (LE) = 55 years
- Per capita gross domestic product (Int$ GDPPC) = Int$ 2826.788
- Per capita total expenditure on health (PCTHE) = Int$ 177.3455
- NHGDPPC = GDPPC − PCTHE = Int$ 2826.788 – Int $177.3455 = Int$ 2649.4425
- Discount rate ( r ) = 3 %
- Undiscounted years of life lost under 5 years (YLL ) = LE – AAD – 14 years = 55 – 2.5 – 14 = 38.5 years
- Discounted years of life lost (DYLL ) = 22.80821513
- NHGDPLoss = DYLL x Int$ NHGDPPC x TCD = 22.80821513 x 2649.4425 x 804000 = Int$ 48 584 959 830

The above formulas were built in Excel software to avoid errors. The non-health GDP losses for the remaining 46 countries in the African Region were estimated in a similar manner.

### Data sources and analysis

The data on life expectancy at birth, total child deaths, per capita GDP in purchasing power parity (PPP) and per capita total health expenditure for each of the 47 countries in the African Region were obtained from the World Health Statistics 2015 [1].

The algorithm used in estimation of non-health GDP losses (equation 1) was built in an Excel spreadsheet. In order to facilitate comparison for the analysis, the countries were put into three economic groups as shown in *Table*1, with high and upper middle income countries in Group 1, lower middle income countries in Group 2 and low income countries in Group 3.

**Table 1:** Economic classification of WHO African Region countries in 2013

Group	GNI per capita (US$)	Countries
Group 1: High income and upper middle income	>= 4086	Algeria, Angola, Botswana, Equatorial Guinea, Gabon, Mauritius,^a^ Namibia, Seychelles,^a^ South Africa (9)
Group 2: Lower middle income	1036–4085	Cameroon, Cape Verde,^a^ Congo, Cote d’Ivoire, Ghana, Kenya, Lesotho, Mauritania, Nigeria, Sao Tome and Principe,^a^ Senegal, Swaziland, Zambia (13)
Group 3: Low income	1036 or less	Benin, Burkina Faso, Burundi, Central African Republic, Chad, Comoros, Democratic Republic of Congo, Eritrea, Ethiopia, Gambia, Guinea, Guinea-Bissau, Liberia, Madagascar, Malawi, Mali, Mozambique, Niger, Rwanda, Sierra Leone, South Sudan, Togo, Uganda, United Republic of Tanzania, Zimbabwe (25)

Notes: ^a^Since no child deaths were reported from Cape Verde, Mauritius, Seychelles and Sao Tome and Principe, those countries were not included in the analysis

### Ethical clearance

This study is entirely an analysis of data from published secondary sources. Since human subjects were not involved, it did not require ethical clearance.

## Results

The WHO African Region’s population and child deaths by economic group in 2013 are presented in *Table*2. Out of the total of 2 976 000 child deaths that occurred, 7.9 % were borne by the high and upper middle income countries (Group 1), 41.26 % by the lower middle income countries (Group 2) and 50.84 % by the low income countries (Group 3). The average number of child deaths per country was 64 696 (STD = 126 062) with a wide variation, ranging from 0 in Cape Verde, Mauritius, Sao Tome and Principe and Seychelles to 804 000 in Nigeria. The regional average life expectancy at birth was 60 years (STD = 6), with a minimum of 46 years in Sierra Leone and a maximum of 75 years in Cape Verde. The average non-health GDP per capita in the Region was Int$ 4171.6 (STD = 5996.8), varying from Int$ 382 in the Democratic Republic of Congo to Int$ 25 878 in Seychelles. The regional average total health expenditure was Int$ 246 (STD = 339) with a minimum of Int$ 19 in Eritrea and a maximum of Int$ 1503 in Equatorial Guinea.

**Table 2:** Population and child deaths by economic group in WHO African Region countries in 2013

Group/economic class	Population	Child deaths
Group 1: High income & upper middle income	120 209 000	235 000
Group 2: Lower middle income	331 277 000	1 228 000
Group 3 : Low income	478 356 000	1 513 000
Grand total	929 842 000	2 976 000

Source: WHO [1]

Note: Since no child deaths were reported from Cape Verde, Mauritius, Seychelles and Sao Tome and Principe, those countries’ population figures are not included

### Non-health GDP loss attributable to child deaths

The 2.976 million child deaths that occurred in the African Region in 2013 could potentially decrease future non-health GDP by Int$ 150 269 716 211 (*Table*3). Approximately 27.3 % of the loss would be borne by Group 1 countries, 47.1 % by Group 2 and 25.7 % by Group 3. The average total non-health GDP loss would be Int$ 50 494 per child death. The expected non-health GDP loss across the Region would vary widely, from Int$ 0 in Cape Verde, Mauritius, Sao Tome and Principe and Seychelles to Int$ 48.6 billion in Nigeria. The reader should bear in mind that the amounts reported in this paper reflect the potential loss of future discounted non-health GDP likely to accrue from premature mortality of under-fives.

**Table 3:** Discounted values of future non-health GDP losses from under-five child deaths among WHO African Region countries in 2013 (Int$ or PPP)

Countries	International dollars (PPP)
Algeria	4 732 018 441
Angola	20 370 636 893
Benin	1 145 975 018
Botswana	779 331 330
Burkina Faso	2 150 494 988
Burundi	478 028 184
Cameroon	4 028 162 632
Cape Verde	-
Central African Republic	310 887 770
Chad	4 476 126 613
Comoros	60 815 079
Congo	901 061 974
Cote D’Ivoire	2 659 062 676
DRC	2 494 491 554
Equatorial Guinea	1 120 718 974
Eritrea	190 689 672
Ethiopia	6 414 441 094
Gabon	1 416 228 173
Gambia	273 278 235
Ghana	5 304 672 102
Guinea	1 048 730 163
Guinea Bissau	185 155 327
Kenya	4 599 055 489
Lesotho	253 071 356
Liberia	148 849 331
Madagascar	995 663 261
Malawi	769 215 438
Mali	2 011 371 453
Mauritania	625 063 654
Mauritius	-
Mozambique	2 164 395 453
Namibia	590 738 828
Niger	1 643 370 477
Nigeria	48 584 960 538
Rwanda	777 510 502
Sao Tome & Principe	-
Senegal	1 437 227 290
Seychelles	-
Sierra Leone	815 282 487
South Africa	11 953 182 706
South Sudan	1 927 355 917
Swaziland	349 080 230
Tanzania	3 792 758 053
Togo	506 247 096
Uganda	3 335 104 652
Zambia	1 972 321 864
Zimbabwe	476 883 244
Total loss (Int$)	150 269 716 211

#### Group 1 countries’ non-health GDP loss

**Fig. 3:**
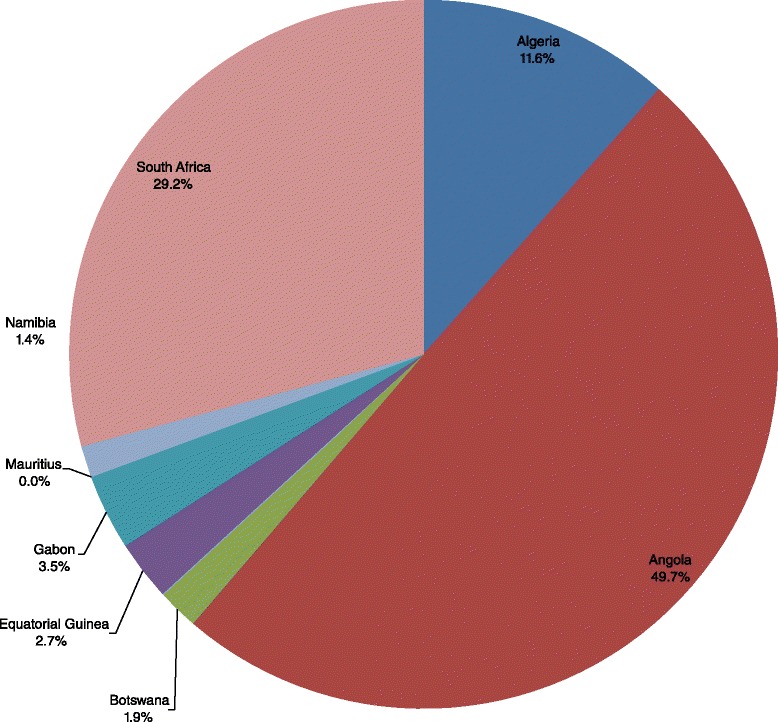
Group 1’s non-health GDP loss due to child deaths in high income and upper middle income countries of WHO African Region, 2013

The 235 000 child deaths in Group 1 countries resulted in an expected total loss of Int$ 40 962 855 345 in non-health GDP in 2013, which was equivalent to 3.68 % of the group’s total GDP. The total productivity loss varied greatly, from Int$ 0.591 billion in Namibia to Int$ 20.4 billion in Angola. Figure 3 shows the distribution of Group 1’s total non-health GDP loss across the eight high and upper middle income countries. About 49.7 % of the loss was borne by Angola.

#### Group 2 countries’ non-health GDP loss

**Fig. 4:**
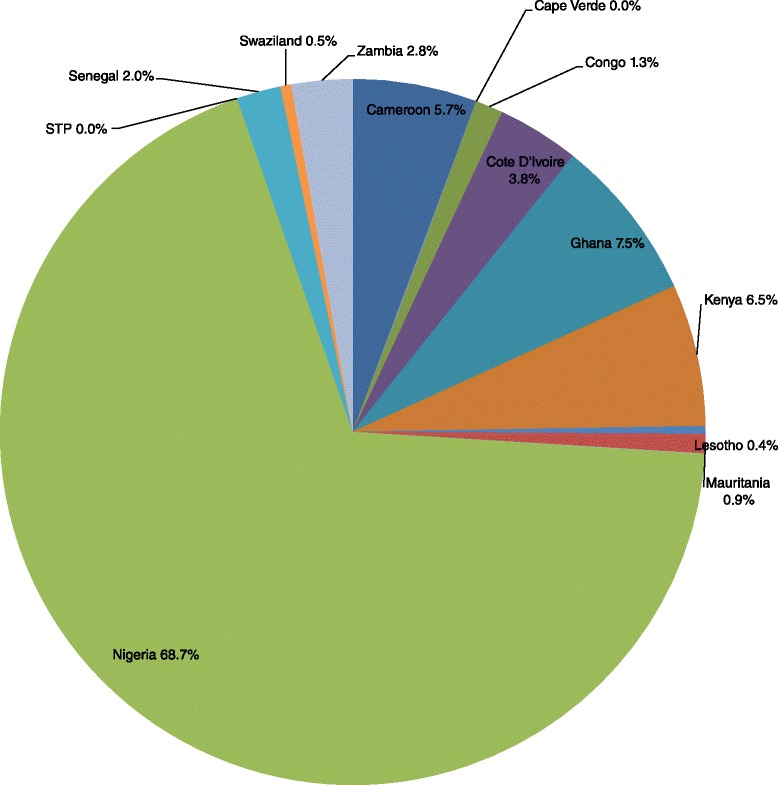
Group 2’s non-health GDP loss due to child deaths in lower middle income countries of the WHO African Region, 2013

The 1 228 000 child deaths in Group 2 countries resulted in an expected total loss of Int$ 70 713 739 806 in non-health GDP in 2013, or 8.4 % of the group’s total GDP. The loss ranged from Int$ 0 in Cape Verde and Sao Tome and Principe to Int$ 48.6 billion in Nigeria. Figure 4 shows the distribution of Group 2’s total non-health GDP loss across the 12 lower middle income countries. Approximately 68.7 % of Group 2’s expected loss was borne by Nigeria.

#### Group 3 countries’ non-health GDP loss

**Fig. 5:**
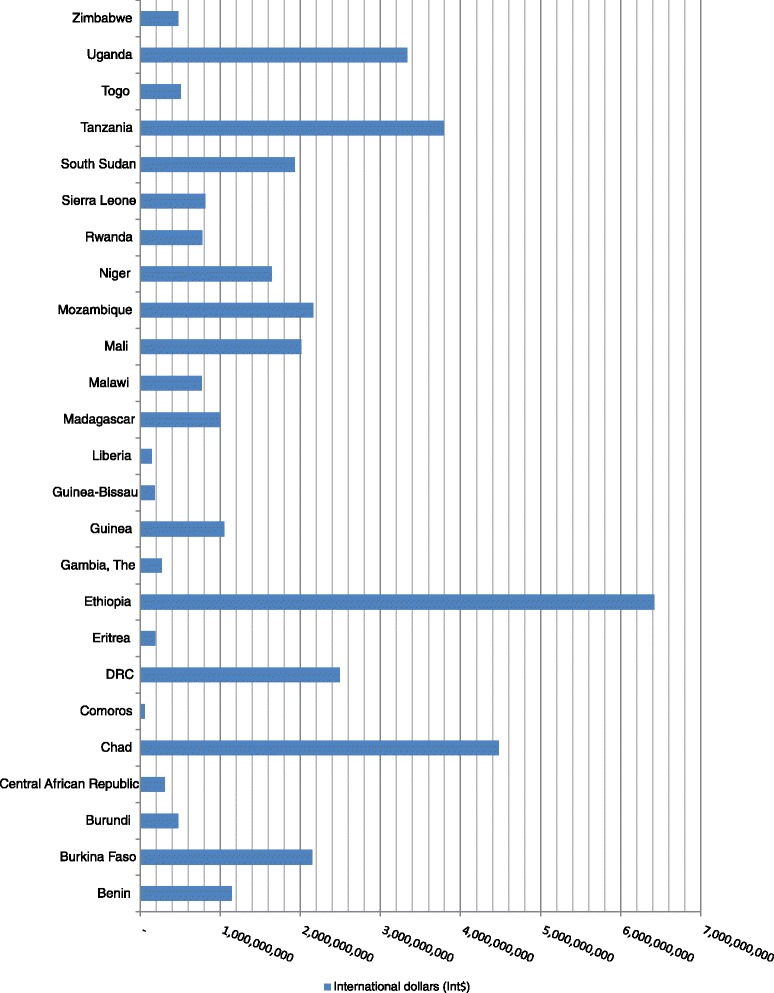
Group 3’s non-health GDP loss due to child deaths in low income countries of the WHO African Region, 2013

Some 1 513 000 child deaths occurred in Group 3 in 2013 and resulted in a total expected loss in non-health GDP of Int$ 38 593 121 061, which is equivalent to 7.13 % of the group’s total GDP. The expected loss ranged from Int$ 60.8 million in Comoros to Int$ 6.4 billion in Ethiopia, which bore 16.6 % of the group’s loss. The distribution of Group 3’s total non-health GDP loss across the 25 low income countries is depicted in Fig. 5. Chad, Ethiopia, Mozambique, Tanzania and Uganda combined accounted for 52.3 % of the expected loss in this group. Even though Group 3 had 285 000 more child deaths than Group 2, the non-health GDP loss of Group 2 was higher than that of Group 3 by Int$ 32.12 billion because Group 2 had higher per capita GDP.

#### Average GDP losses

The average non-health GDP losses per child death and per person in the population for the 47 countries are portrayed in *Table*4. These values were obtained by dividing a group’s total productivity loss by its total child deaths. The average non-heath GDP loss per person in the population for each group was calculated by dividing the group’s total GDP loss by its population (*see Table*2).

**Table 4:** Discounted values of future non-health GDP lost due to child deaths in 2013 by economic group

Cost item	Group 1 (Int$)	Group 2 (Int$)	Group 3 (Int$)	Total cost (Int$)
Total cost of child deaths	40 962 855 345	70 713 739 806	38 593 121 061	150 269 716 211
Average cost per child death	174 310	57 584	25 508	50 494
Average cost per person in population	340.8	213.5	80.7	161.6
% of Grand Total	27.3	47.1	25.7	100.0

The average non-health GDP lost per child death was Int$ 174 310 for Group 1, Int$ 57 584 for Group 2 and Int$ 25 508 for Group 3. The average non-health GDP loss per person in the population was Int$ 341 for Group 1, Int$ 213 for Group 2 and Int$ 81 for Group 3. The average non-health GDP lost per child death in Group 1 was about three times that for Group 2 and almost seven times that for Group 3.

The main determinant of expected productivity loss is the magnitude of per capita GDP. For instance, even though child deaths in the middle income countries like Botswana, Equatorial Guinea, Gabon and Namibia totalled only 2000, 2000, 3000 and 3000, respectively, the non-health GDP losses per child death for these countries were substantial at Int$ 389 666 for Botswana, Int$ 560 359 for Equatorial Guinea, Int$ 472 076 for Gabon and Int$ 196 913 for Namibia. Low income countries with relatively high total child deaths such as for the Democratic Republic of Congo with 320 000 deaths, Ethiopia with 196 000 deaths, Uganda with 102 000 deaths, Tanzania with 95 000 deaths and Niger with 86 000 deaths have comparatively low productivity losses per child death of Int$ 7795, Int$ 32 727, Int$ 32 697, Int$ 39 924 and Int$ 19 109, respectively.

## Discussion

The estimated total expected non-health GDP loss ascribed to child deaths of Int$ 150.3 billion is about 6 % of the combined 2013 GDP of the 47 African countries [37]. This estimate denotes the expected loss in potential GDP in the future from the 2 976 000 child deaths revalued relative to the base year 2013, i.e. present values. The use of forgone future earnings assumes that changes in child mortality rates are reflected in changes in future earnings and national income (as measured by GDP). This assumption may not always hold because such estimates are influenced by a number of transient factors such as distribution of income, education and employment opportunities. This means that a reduction in child mortality may not necessarily translate into increases in GDP. Thus, the expected loss of Int$ 150.3 should be viewed as an estimate of the economic value of lives lost due to premature mortality; and not an indicator of resources that would be saved if those lives were saved.

### Sensitivity analysis

We applied a discount rate of 3 % because it was used also in the WHO health systems’ performance assessment [38], the global burden of disease studies [39], the Institute for Health Metrics and Evaluation’s global burden of disease studies [40] and the economic evaluation studies on health interventions in Africa [41]. Nevertheless, to test the effect of the discount rate on the total expected non-health GDP loss estimate, a one-way sensitivity analysis was conducted at 5 % and 10 % discount rates. Using a 5 % discount rate reduced the total expected non-health GDP loss by Int$ 39.3 billion (26 %) and the average non-health cost per child death by Int$ 13 193, whilst application of the 10 % discount rate decreased the grand total non-health GDP loss by Int$ 87.2 billion (58 %) and the average non-health cost per child death by Int$ 29 316. This signifies that the magnitude of the total economic loss is partially dependent on the discount rate utilized.

We used 2.5 years (a simple average) as the average age at death. This value was used owing to the lack of data on age distribution of child deaths. Nonetheless, since the distribution of child deaths is unlikely to be uniform over the 0–5 year range, a sensitivity analysis was conducted to determine the effect of age on the total non-health GDP loss estimate. The model was first re-estimated assuming an average age at death of 0 years. The utilization of this value raised the total non-health GDP loss by Int$ 3.7 billion, a 2.5 % increase.

The model was re-estimated assuming an average age at death of 5 years. This average reduced the total non-health GDP loss by Int$ 5.98 billion, a 4 % decrease. This implies that the magnitude of the expected non-health GDP loss to a limited extent also depends on the average age used for the onset of child deaths. Therefore, there is need for more investments in research to come up with reliable data on age distribution of child deaths in Africa.

### Implications

To a large extent child morbidity and deaths and the associated microeconomic and macroeconomic losses could be prevented if all children had unfettered access to the available and cost-effective newborn, infancy and childhood interventions [42, 43]. WHO provides details on the packages of interventions essential for children for the home or community level, and primary level and referral health facilities, and which, if implemented to scale, could end preventable child deaths [44]. Over a decade and half ago, WHO and United Nations Children’s Fund (UNICEF) published a document presenting an integrated approach to improving management of childhood illnesses, which is still effective [45].

For childhood interventions to be effectively and efficiently delivered in an integrated manner to the needy population groups, the national and local health systems need to be strengthened to become resilient to shocks of whatever kind [46, 47]. That entails programmatic leadership and governance to plan, guide, support, monitor and evaluate health promotion and service delivery within the model of a continuum of care, where the health services are always available, accessible, safe and acceptable; the health workforce is of adequate numbers and mix and has the required range of competencies; life-saving supplies and commodities are available; technology is up to date; the health financing system covers health promotion and services for pregnant women, newborns, infants and children; and health management information systems are effective [48].

There is need for investments in other sectors to adequately address socioeconomic determinants of health, including building or strengthening relevant structures to ensure that civil and vital registration systems that facilitate tracking of child births, mortality and causes of death are functional [49] and strengthening national health research systems to promote the generation and use of epidemiological and clinical research, social-cultural and behavioural change research, implementation research, and health systems and economic research [50–53]. Similarly, human rights tools and frameworks will need to be strengthened to achieve better outcomes, to apportion accountability for women’s and children’s health, and to institutionalize maternal, newborn and child mortality censuses [13, 48].

### Limitations of the study

Cost-of-illness studies like the one reported in this paper strictly are not meant to inform public health priority setting because they do not compare the costs and consequences of alternative interventions that could prevent child morbidity and mortality [54, 55]. Therefore, the purpose of our study was not to guide priority setting but rather to raise awareness of the public and policy-makers in the ministries of health and finance on the negative impact of child deaths on non-health GDP.

The study did not include direct health-care costs such as those related to vaccines, drugs, tests, supplies, hospital personnel, diagnostic equipment and physical facilities; direct non-health-care costs of treatment such as transport to and from the health service provider; patient time costs for treatment such as those relating to travel and waiting and treatment time; cost of the time informal caregivers, volunteers, family or friends spend accompanying or visiting the sick person; loss in productivity due to morbidity; or intangible costs such as pain and grief [56, 57].

The analysis reported in this paper is based on estimates of under-five mortality reported in the *World Health Statistics 2015* [1]. Those estimates are derived wherever possible from death registration data reported annually to WHO. Unfortunately, very few African Region countries have civil registration and vital statistics systems (CRVS) that permit adequate and regular tracking of mortality and causes of death [49]. For instance, out of the 46 WHO African Region Member States in 2007, only Algeria, Mauritius, Seychelles and South Africa had a death registration coverage rate of 75 % or higher [58]. For countries where such data are not available or are of poor quality, WHO uses household surveys (for births and child deaths) and censuses to prepare estimates of mortality rates and life expectancy. As AbouZahr et al. [59] eloquently state, the need for support to countries to develop functional CRVS and to institutionalize international classification of diseases cannot be overemphasized.

## Conclusions

The limitations of this study notwithstanding, the heavy economic burden of child deaths and human rights concerns call for urgent acceleration of action by governments, the private sector, the civil society and development partners to fully implement the letter and spirit of decisions and resolutions on child health from the African Union [11–16], the World Health Assembly [7–10] and the United Nations [2–4]. Governments and the private sector in the Region, along with their development partners, failed the children who died prematurely due to the failure to provide the required investments for full realization of the health-related MDGs. These institutions will be judged harshly by history should they fail once more to fulfil the post-2015 health Sustainable Development Goal 3 on ensuring healthy lives and promoting well-being for all at all ages.

Therefore, governments, the private sector and development partners should in solidarity sustainably and equitably provide the resources necessary to build resilient national health systems and structures affecting the determinants of health to facilitate the provision of basic needs such as clothing, education, food, shelter, clean sanitation and water, to end preventable child morbidity and mortality [60].

## Abbreviations

AAD: average age at death
DPT3: a third dose of vaccine against diphtheria, pertussis and tetanus
DYLL: discounted years of life lost
GDP: gross domestic product
GDPPC: per capita GDP
HepB3: third doses of hepatitis B vaccine
Hib3: 3 doses of haemophilus influenzae type b vaccine
ILO: International Labour Organization
Int$: International dollars
ITN: insecticide treated net
LE: Country’s average life expectancy at birth
MDGs: Millennium Development Goals
MTCT: prevention of mother-to-child transmission of HIV
*n*: final year of the total number of years of life lost per child death
*NHGDPPC*_*Int$*_: per capita non-health gross domestic product in purchasing power parity (PPP)
NHGDPLoss: non-health GDP loss
PCTHE: Per capita total health expenditure
PPP: purchasing power parity
*r*: rate of discount of future losses
SDG: sustainable development goals
STD: Standard deviation
*t*: First year of life lost
*TCD*: total number of child deaths
UNICEF: United Nations Children’s Fund
WHO: World Health Organization
YLL: undiscounted years of life lost
